# IMPROVING HOSPITAL AND POST-ACUTE OUTCOMES AND EXPERIENCES OF DYADS LIVING WITH DEMENTIA: RESULTS OF THE FAM-FFC TRIAL

**DOI:** 10.1093/geroni/igad104.0300

**Published:** 2023-12-21

**Authors:** Marie Boltz, Ashley Kuzmik, Karen Rose

## Abstract

Hospitalizations of persons living with dementia are associated with protracted functional decline, delirium, and behavioral symptoms of distress as well as increased care partner stress. This symposium presents findings from the Family-centered Function -focused Care (Fam-FFC) trial. In Fam-FFC, nurses purposefully empower family care partners in the assessment, decision-making, care delivery, and evaluation of function-focused care during hospitalization and the 60-day post-acute period. The goal is to improve the functional recovery of the patient while increasing family preparedness for caregiving, bridging hospital to post-acute setting. This cluster randomized controlled trial tested the impact of Fam-FFC in 455 dyads of diverse persons living with dementia and their care partners. The first presentation will compare Fam-FFC outcomes for patients and care partners to the attention control group outcomes at discharge, two and six months. The second presentation will report the impact of Fam-FFC upon adverse patient events (falls, emergency department transfers, hospital admissions) in the two arms. Next, the presenter will utilize Fam-FFC data to describe the patient and care partner characteristics that contribute to care partner distress at the time of hospital discharge. The fourth presentation will focus on staff-patient interactions in the Fam-FFC trial, including the influence of clinical factors upon the quality of the interaction. The final paper will report on the trajectory of behavioral symptoms of distress, and associated outcomes, from hospital admission to six-month follow-up. Our discussant, Karen Rose will lead a discussion of unanswered questions, next steps, and implications of this work for policy and practice.

